# HEALTH CARE PROVIDER ADOPTION OF COGNITIVE ASSESSMENT AND CARE PLAN SERVICES FOR PATIENTS LIVING WITH DEMENTIA

**DOI:** 10.1093/geroni/igae098.2978

**Published:** 2024-12-31

**Authors:** John Mulcahy

**Affiliations:** University of Minnesota, Minneapolis, Minnesota, United States

## Abstract

In 2017 the Centers for Medicare and Medicaid Services introduced a new billable code for Cognitive Assessment and Care Plan (CACP) Services to encourage healthcare providers to take a more active role in identifying their patients who are living with Alzheimer’s Disease and Related Dementias (ADRD), and to work with these patients and their care partners to develop a comprehensive long-term care plan. This CACP service has seen very low adoption with 0.5% of Medicare beneficiaries with an ADRD diagnosis having received CACP in 2017 and only 2.9% of beneficiaries with ADRD having received it by the end of 2020. Receipt of CACP was even more dismal among beneficiaries living with ADRD who are Hispanic/Latino, dual-eligible, or living in a rural county with 1.8%, 1.9%, and 1.8% of these beneficiaries receiving CACP by 2020 respectively. It is unknown if there are specific barriers to adoption driving this low uptake. This analysis uses a 100% samples of Medicare fee-for-service claims and Medicare Advantage encounters to identify provider and practice level predictors correlated with CACP adoption. The model predicts adoption of CACP using provider specialty, peer adoption of CACP, a previously denied CACP claim, number of patients living with ADRD treated by the provider, percent of patients in Medicare Advantage, overall patient volume, and practice size. Understanding the relative contribution of these potential barriers/facilitators to provider adoption will help inform dissemination efforts and changes that can be made to billing requirements to encourage widespread CACP uptake.

